# The Effect of Smoke-Free Air Law in Bars on Smoking Initiation and Relapse among Teenagers and Young Adults

**DOI:** 10.3390/ijerph120100504

**Published:** 2015-01-09

**Authors:** Ce Shang

**Affiliations:** Institute for Health Research and Policy, University of Illinois at Chicago, Chicago, IL 60608, USA; E-Mail: cshang@uic.edu; Tel.: +1-312-996-0774; Fax: +1-312-996-2703

**Keywords:** smoke-free air laws in bars, smoking initiation, smoking relapse, longitudinal study, cigarette taxes, NLSY97, youth and young adults, hazard model

## Abstract

*Background*: Existing evidence has shown that most smoking uptake and escalation occurs while smokers are teenagers or young adults. Effective policies that reduce smoking uptake and escalation will play an important role in curbing cigarette smoking. This study aims to investigate the effect of smoke-free air (SFA) laws in bars on smoking initiation/relapse while controlling for other confounders. *Methods*: The national longitudinal Survey of Youth 1997 (NLSY97) from 1997–2009 was linked to state-level scores for the strength of SFA laws in order to carry out the analysis. *Results and Conclusion*: We find that SFA laws in bars with exemptions significantly reduce (*p* ≤ 0.01) the probability of smoking initiation (one-puff, daily, and heavy smoking initiation). The 100% SFA law in bars without exemption significantly deters smoking relapse from abstinence into daily smoking (*p* ≤ 0.05) or relapse from abstinence into heavy smoking (*p* ≤ 0.01) among people age 21 or older. The reduction of one-puff and daily smoking initiation is larger among ages 20 or younger than ages 21 or older, while the reduction in relapse does not differ by whether respondents reach the drinking age. Results also indicate that higher cigarette taxes significantly reduce daily smoking initiation and relapse into nondaily and light smoking.

## 1. Introduction

Existing evidence suggests that smoking initiation often occurs at an early age and develops into regular smoking through dependence or addiction later [1,2,3,4,5,6]. Therefore, effective tobacco control policies that deter smoking initiation and escalation are crucial in curbing tobacco use. However, current evidence of the effectiveness of policies including price/tax and other tobacco control laws is best described as mixed. For example, increasing cigarette taxes or prices are found to significantly deter smoking initiation in some studies [6,7,8,9,10,11] but not in others [12,13,14,15]. Moreover, studies of the effects of smoke-free air (SFA) laws on reducing smoking initiation and escalation are very limited and almost all supporting evidence is suggestive [16,17,18,19,20,21].

In particular, among all the venues where SFA laws are imposed, SFA laws in bars merit more attention regarding their potential impacts on smoking initiation and escalation. This is because, first, SFA laws in workplaces, bars, and restaurants have been the center of state and local SFA legislations [16,17], and, second, substantial evidence has shown that, similar to smoking initiation, drinking also initiates at an early age and escalates in young adulthood [22,23,24,25,26,27]. Given that a significant number of underage drinking happens at bars [26,28], and that smoking and drinking are complementary risky behaviors [29,30,31,32,33], SFA laws in bars may play a more important role in deterring smoking initiation and escalation compared with SFA laws in other venues. Third, there is lack of evidence on whether the effectiveness of SFA laws in bars varies by whether people have reached legal drinking age [34].

In sum, current research on SFA laws in bars have been focused on justifying that these laws have no economic consequences on the revenues of the affected bars [35,36,37] and their effects on reducing secondhand smoking exposure [34,38,39,40,41,42]. Research is regarding on the effectiveness of these laws on reducing smoking initiation/escalation and whether their impacts on smoking behaviors differ by legal drinking status is clearly needed. Moreover, given SFA laws in bars are shown to correlate with less drinking [31,32,33] and less secondhand smoke exposure [38,39,40,41,42], such studies will add evidence to the potential significant public health impact of SFA laws in bars.

This study will utilize the National Longitudinal Survey of Youth 1997 (NLSY97) from 1997–2007 to analyze the impact of SFA laws in bars on smoking initiation and relapse into multiple levels of smoking. The NLSY 97 is particularly suitable to conduct such a study as it has run parallel with the period during which states gradually adopted SFA laws or more restrictive SFA laws [43]. In addition, NLSY97 is longitudinal data and, unlike a synthetic panel created using retrospective information in cross-sections, it has the following advantages in studying smoking initiation/relapse: (1) the actual transitions were observed and are less likely to contain recall errors; (2) the state of residence was tracked over years and is less likely to induce measurement errors in the linkage to policy data; (3) time-variant socioeconomic characteristics such as marital status can be controlled.

We find that SFA laws in bars with exemptions significantly reduce smoking initiation (one-puff, daily smoking initiation, heavy smoking initiation) among all ages and smoking relapse into non-daily smoking among ages 20 or younger. The 100% SFA law in bars without exemption significantly deters smoking relapse from abstinence into daily and heavy smoking among those aged 21 or older. The reduction of initiation is larger among people aged 20 or younger than those aged 21 or older, while the reduction in relapse does not differ by whether respondents reach the drinking age. Results also indicate that higher cigarette taxes significantly reduce daily smoking initiation and relapse into nondaily and light smoking.

## 2. Methods

### 2.1. NLSY97

In this study, we used National Longitudinal Survey of Youth 1997 (NLSY97) from 1997 to 2009 to identify smoking initiation/relapse and to conduct the analysis. NLSY97 is a longitudinal survey that tracks a nationally representative sample of 8984 youth who were 12–16 years old as of 31 December 1996 over time. Therefore, the age range for NLSY97 during 1997–2009 is 12–30 years. Given that respondents were interviewed on an annual basis, smoking initiation/relapse can be identified through comparing self-reported smoking status in each year. For example, initiation occurred when respondents who had never smoked by the previous year’s survey reported to have smoked in the past 30 days in this year’s survey. Moreover, smoking status is reported both in frequency (During the past 30 days, on how many days did you smoke a cigarette?) and in intensity (When you smoked a cigarette during the past 30 days, how many cigarettes did you usually smoke each day?), which allows us to categorize smoking initiation/relapse into four distinguishable stages: (1) transition into non-daily smoking; (2) transition into daily smoking; (3) transition into light smoking(<10 cigarettes per day); and (4) transition into heavy smoking (≥10 cigarettes per day).

In addition to smoking status, NLSY contains demographics of respondents and tracks their socioeconomic characteristics over years. The demographic and socioeconomics controls we used in the analysis are gender, race/ethnicity (non-Hispanic White-referent, Hispanic, non-Hispanic Black, and non-Hispanic other races), employment status (worked in employee-type job since last interview *vs.* not), marital status (married *vs.* not), and education/school enrollment levels constructed using enrollment status and highest degree received (college degree or higher-referent, enrolled in college, high school degree not enrolled in college, enrolled in high school, and high school dropout). Because the effects of time-variant socioeconomic variables on smoking initiation/relapse are likely to differ by youth and young adults (e.g., being single may have very different impacts on smoking among teenagers than among young adults), we also control for interactions of these socioeconomic variables and a dichotomous variable for being younger than age 21.

Furthermore, NLSY97 Geocode file contains state of residence for each respondent in each year that allows for a linkage to state-level cigarette taxes, smoke-free policies, and other controls. After stacking NLSY97 data from 1997–2009, we dropped those who did not report smoking status or whose state of residence were missing, which combined were about 13% of the sample. This attrition rate is similar to the one reported in Aughinbaugh and Gardecki (2007) [44]. For the missing marital or education/enrollment status, we compared the non-missing values in the preceding and following years and, if they are the same or can be inferred (e.g., missing grade between 10 grade in the preceding and 12 grade in the following year would be filled with 11 grade), we replaced missing values with the non-missing or inferred values; otherwise, we replaced the missing values using the non-missing values in the preceding year. Sensitivity analyses by dropping these missing covariates were also conducted.

### 2.2. State-Level SFA Laws, Cigarette Taxes, and Other Controls

**Figure 1:**
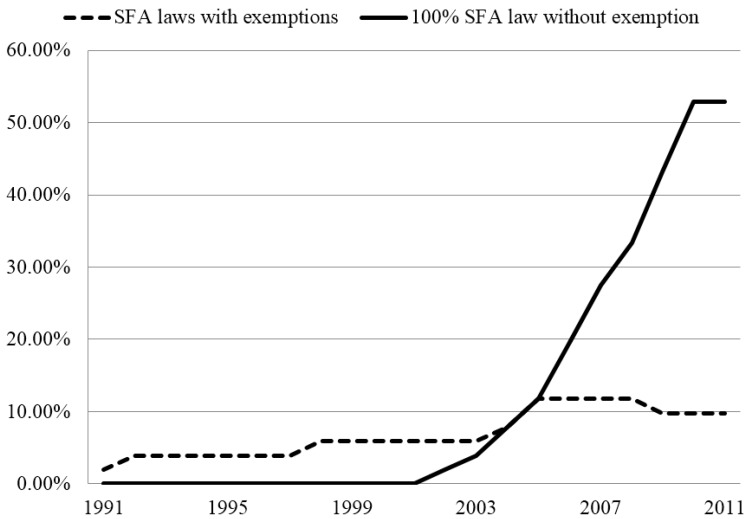
The Prevalence of smoke-free air (SFA) Laws in Bars in 50 States and DC.

The SFA laws in 13 venues, state tobacco control funds in 1982–1984 dollars, and the number of youth access laws prohibiting minors from possessing, using, and purchasing tobacco products (PUP) for each state and year during 1997–2009 were obtained from Impacteen. The SFA venues include bars, restaurants, private work places, public work places, public transit, recreational centers, *etc.* For each venue, the strength of SFA laws is rated on a scale of 0–5 or 0–3 annually for each state, which leads to a total score of 49. For restaurants (including bar areas of restaurants) and bars, where 0 equates no laws, 1–2.5 equates SFA laws with exemptions such as designated smoking areas or ventilation standards, and 3 equates 100% smoke free, no exemption. Figure 1 shows, over years, the percentages of states having any SFA laws with exemptions and states having the 100% SFA law with no exemption in bars during 1997–2009. Most states imposed SFA laws with certain exemptions in earlier years and had gradually moved towards 100% SFA law without exemptions.

In order to fully study the effect of SFA in bars, we created dichotomous indicators for different strengths of laws (none-referent, SFA laws with exemptions, and 100% SFA law with no exemption). Because the legal drinking age in the US is 21, we also included interaction terms for the two law indicators and a dichotomous indicator of being younger than 21 and controlled for them in the analyses. The SFA law in restaurants measured using a 0–3 scale is also controlled in all analyses. Although in most previous studies, SFA laws in restaurants and bars are studied/considered the same, the data show that about 46% of the states in the study period have imposed a different SFA law in bars than in restaurants. Therefore, it is important to study the effects of SFA laws in bars and in restaurants separately. In addition to SFA laws in bars and restaurants, laws in other venues were controlled using an average SFA policy index, which was constructed using the sum of the scores of the rest 11 venues divided by the highest possible total score of 43. In a separate sensitivity check, we controlled for individual SFA law scores of these 11 venues instead of the average SFA policy index.

State-level average cigarette taxes for the calendar year were obtained from the annual *Tax Burden on Tobacco* [45] and include the average federal tax of the year. We converted the taxes into 2009 dollars using the Consumer Price Index from Bureau of Labor Statistics. Finally, we merged SFA laws, state tobacco control funds, PUP index, and average cigarette taxes to the NLSY97 data using the state identifier and year.

### 2.3. Methodology

The Discrete-time Hazard Model is employed to analyze smoking initiation (one-puff, daily-smoking initiation, and heavy-smoking initiation) and relapse (relapse to nondaily/daily smoking and relapse to light/heavy smoking). This method has been widely used in analyzing smoking dynamics in the literature [46,47] and will estimate the impact of explanatory variables on the “hazard” probability of the outcome. In order to identify the model, all individuals start from a baseline status (outcome = 0) and are followed over time. Once an individual transits into the studied status (outcome = 1), the “hazard” occurs and he or she will be subsequently dropped out of sample. Those who remained at the baseline status 0 and had never made the transition by the end of the study period will be kept in the sample at all times. After the data is shaped into such format, logistic regressions are usually used for estimation. Additionally, this model can be extended to the Competing Risk Model that estimates transitions into two or more “competing” outcome statuses, and analyzed using multinomial regressions. The following equation shows the Competing Risk model with two competing or mutually exclusive outcome status (outcome = 1 or 2) that are different from baseline 0.

(1) $$\mathrm{Pr}\left(Y_{t}=j|Y_{t-1,..1}=0\right)=\frac{e^{X_{it}\beta_{j}}}{\sum_{j=0}^{2}e^{X_{it}\beta_{j}}}\;,\;j=0,\;1,\;2,\;\overset{2}{\underset{j=0}{\sum }}\text{Pr}(Y_{t}=j)=1\;\;\;\;\;\;$$

More specifically, smoking initiation (one-puff, daily-smoking initiation, and heavy-smoking initiation) was estimated using the Hazard model with one potential risk while smoking relapse (relapse to nondaily/daily smoking and relapse to light/heavy smoking) was estimated using the Competing Risk Model with two competing risk statuses—Those are, in smoking frequency, either relapsing into nondaily or into daily smoking; and in smoking intensity, either relapsing into light or into heavy smoking.

The study samples were obtained in the following ways: (1) for smoking initiation with even one-puff of a cigarette, we first dropped ever-smokers in the baseline year 1997, then assigned never-smokers 0 in the following years until they initiated; (2) for daily-smoking initiation, we first identified non-daily smokers or non-smokers in the baseline year 1997, then assigned non-daily smokers and non-smokers 0 in the following years until they started smoking daily; (3) for heavy-smoking initiation, we first identified light smokers or non-smokers in the baseline year 1997, assigned light smokers and non-smokers 0 in the following years until they started heavy-smoking; (4) for relapsing in smoking frequency, we first identified eve-smokers who were not smoking, took the first year when we observed them to be abstinent (either 1997 or if smokers quit during 1997–2009, the year when they quit) as the baseline year, assigned abstinent non-smokers 0, and followed them until they relapsed to smoking. Then, we coded those who relapsed to non-daily smoking as 1 and those relapsed to daily smoking as 2; 5) for relapsing in smoking intensity, the procedure is the same with the procedure for relapsing in frequency. After identifying relapsing, those who relapsed to light smoking were coded as 1 and those who relapsed to heavy smoking were coded as 2. Because some smokers may report positive smoking intensity with a 0 frequency or vice versa, the samples sizes for two relapsing samples are slightly different.

Finally, the effects of SFA laws in bars (none-referent, SFA laws with exemptions, and 100% SFA law with no exemption) on smoking initiation/relapse and their interactions with age were examined along with state fixed effects, year fixed effects, individual-level socioeconomic and demographic controls and state-level controls described in the data section. In addition, duration dependence that measures how the hazards of outcomes change over time [46,47] is controlled using the natural log form of age since 10. Similar to previous studies [8,9,10,11], because we selected sub-populations who were at risk of making the particular transitions, NLSY weights that adjust for non-responses become inappropriate and thus were not used. For all analyses, standard errors were clustered at the state level. Stata Special Edition 13.1 was used to implement the analyses.

In order to examine the validity of the estimates, sensitivity analyses using different specifications or samples were conducted. These include: (1) instead of year fixed effects, a linear year trend that is less collinear with SFA policies was used; (2) instead of the average SFA policy index, individual SFA policy scores that are less restrictive in modeling the impacts of other policies were estimated with a linear year trend and other covariates; (3) samples were restricted to respondents who reached the drinking age of 21, and 4, samples were restricted to respondents with non-missing covariates.

## 3. Results and Discussion

In Table 1 and Table 2, we present the variable descriptions and summary statistics of the samples used for estimating initiation of even one puff, daily smoking initiation, heavy smoking initiation, relapse into nondaily/daily smoking, and relapse into light/heavy smoking. The summary statistics suggest that in the study period, average probabilities of initiation with one-puff, daily smoking initiation, and heavy-smoking initiation are about 6%, 3%, and 2%, respectively. The probability of smoking relapse (not shown) is about 21% with 15%–16% into nondaily smoking or light smoking and about 5%–6% into daily or heavy smoking. On average, about 18% of the sample is under some SFA laws in bars with some exemptions while about 10% of the sample is under the 100% SFA law in bars without any exemption. The summery statistics for indicators also show the demographic composition of the samples. For example, the statistics in Column 1 show that 48% of the sample was male, 42% of the sample was White, 11% of the sample was married, 31% of the sample was enrolled in high school, *etc.* If we compare statistics for the indicator of age 20 or younger across samples (across columns), the results show that the initiation samples consist of more people age 20 or younger (50%–55%) than do the relapse samples (36%–37%).

**Table 1:** Variable definition, NLSY97, 1997–2009.

Variables	Description
State-level policy and other controls, time-variant	
SFA_bar_law	Indicator equals 1 if the state has SFA laws with exemptions (designated areas or ventilation standards), 0 otherwise.
SFA_bar_ban	Indicator equals 1 if the state has 100% SFA laws with no exemption in bars, 0 otherwise.
SFA_Restaurant	SFA law scores ranging from 0–3 (none–100% SFA law with no exemption).
SFA_Policy_index	Average of SFA law scores in 11 venues divided by the maximum possible score 43, ranges from 0–1.
Cigarette Tax	Average cigarette taxes in 2009 dollars for the calendar year, federal tax included.
PUP Index	Laws prohibit minors from possessing, using and purchasing tobacco: 0–no law, 1–3 = 1–3 PUP laws.
TC Fund	State tobacco control funds in 1982–84 dollars
Individual-level Variables, time-variant	
*Outcome variables*	
Initiation-one puff	Indicator equals 0 if the respondent has never smoked; 1 if starts smoking, even one-puff.
Daily smoking initiation	Indicator equals 0 if the respondent has not smoked daily since 1997; 1 if starts smoking daily.
Heavy smoking initiation	Indicator equals 0 if the respondent has not smoked ≥ 10sticks per day since 1997, 1 if starts smoking ≥ 10 sticks per day.
Smoking relapse into nondaily/daily smoking	Category variables, equals 0 if the respondent remains abstinent; equal 1 if relapses into non-daily smoking; 2 equals if relapses into daily smoking.
Smoking relapse into light/heavy smoking	Category variables, equals 0 if the respondent remains abstinent; equal 1 if relapses into light smoking (<10 sticks per day); 2 equals if relapses into heavy smoking (≥10 sticks per day).
Duration Dependence	The natural log of years since 10 years old, equals ln(Age – 10).
Married	Indicator equals 1 if the respondent is married, 0 otherwise.
Age < 21	Indicator equals 1 if the respondent is younger than 21 years, 0 otherwise.
Employed	Indicator equals 1 if the respondent has worked in employee-type job since last interview, 0 otherwise.
School dropout	Indicator equals 1 if the respondent is a school dropout, 0 otherwise.
Enrolled in high school	Indicator equals 1 if the respondent is enrolled in high school, 0 otherwise.
High school degree	Indicator equals 1 if the respondent has a high school degree and is not in college, 0 otherwise
Enrolled in college	Indicator equals 1 if the respondent is enrolled in college, 0 otherwise
Individual-level Variables, time-invariant	
Male	Indicator equals 1 if the respondent is male, 0 otherwise.
Hispanic	Indicator equals 1 if the respondent is of Hispanic origin, 0 otherwise.
Black	Indicator equals 1 if the respondent is non-Hispanic Black, 0 otherwise
Other	Indicator equals 1 if the respondent is non-Hispanic race other than Black and White, 0 otherwise

**Table 2:** Descriptive statistics for the six smoking transition samples NLSY97, 1997–2009.

Covariates	Initiation			Relapse	
	One-Puff	Daily	Heavy	Nondaily/Daily	Light/Heavy
State-level variables, time variant					
SFA_bar_law	0.179	0.184	0.184	0.182	0.182
	(0.383)	(0.388)	(0.387)	(0.386)	(0.386)
SFA_bar_ban	0.087	0.094	0.096	0.119	0.117
	(0.283)	(0.291)	(0.295)	(0.323)	(0.321)
SFA_restaurant	1.110	1.140	1.145	1.199	1.192
	(1.093)	(1.109)	(1.113)	(1.147)	(1.145)
Policy index	0.418	0.426	0.427	0.442	0.440
	(0.262)	(0.265)	(0.266)	(0.279)	(0.278)
Cigarette Tax	0.534	0.546	0.550	0.576	0.574
	(0.270)	(0.274)	(0.277)	(0.286)	(0.285)
PUP index	1.792	1.812	1.811	1.861	1.857
	(0.972)	(0.961)	(0.964)	(0.932)	(0.933)
TC funding	146,601	147,065	147,065	140,611	141,137
	(207,502)	(201,075)	(199,640)	(181,669)	(183,287)
Individual-level variables, time-variant										
Outcome	0.064	0.027	0.019	0.273	0.247
	(0.245)	(0.163)	(0.137)	(0.572)	(0.519)
Duration Dependence	2.265	2.303	2.312	2.430	2.423
	(0.388)	(0.378)	(0.376)	(0.340)	(0.342)
Married	0.110	0.114	0.114	0.178	0.174
	(0.313)	(0.318)	(0.318)	(0.382)	(0.379)
Age < 21	0.546	0.502	0.492	0.363	0.372
	(0.498)	(0.500)	(0.500)	(0.481)	(0.483)
Employed	0.757	0.775	0.777	0.817	0.816
	(0.429)	(0.418)	(0.416)	(0.387)	(0.387)
School Dropout	0.061	0.069	0.073	0.105	0.105
	(0.238)	(0.254)	(0.260)	(0.306)	(0.306)
Enrolled in high school	0.308	0.268	0.259	0.156	0.161
	(0.462)	(0.443)	(0.438)	(0.363)	(0.368)
High school degree	0.165	0.177	0.184	0.241	0.239
	(0.371)	(0.382)	(0.388)	(0.427)	(0.427)
Enrolled in college	0.247	0.248	0.244	0.221	0.221
	(0.431)	(0.432)	(0.430)	(0.415)	(0.415)
College or higher	0.219	0.237	0.239	0.278	0.274
	(0.414)	(0.425)	(0.427)	(0.448)	(0.446)
Male	0.476	0.484	0.484	0.487	0.489
	(0.499)	(0.500)	(0.500)	(0.500)	(0.500)
White	0.422	0.426	0.425	0.480	0.479
	(0.494)	(0.494)	(0.494)	(0.500)	(0.500)
Hispanic	0.226	0.234	0.231	0.244	0.245
	(0.418)	(0.423)	(0.422)	(0.429)	(0.430)
Black	0.319	0.306	0.308	0.251	0.250
	(0.466)	(0.461)	(0.462)	(0.434)	(0.433)
Other	0.033	0.035	0.036	0.025	0.025
	(0.179)	(0.183)	(0.186)	(0.157)	(0.157)
N	38,988	48,271	50,366	27,001	27,927

Note: Variable definitions are in Table 1. Means and corresponding standard deviations (in parentheses) are presented. Summary statistics of state fixed effects, year fixed effects, and interaction terms of age 21 indicator and time-variant individual-level variables are not shown, which may be sent by request.

The odds ratio estimates of the impacts of smoke-free policies in bars on smoking initiation/relapse and their interactions with age are shown in Table 3. The corresponding marginal effect estimates that show the unit-change in outcomes due to a change from no laws to SFA laws were also reported for respondents aged ≥ 21 and aged < 21, respectively. We find that SFA laws in bars with some exemptions significantly reduce (*p* ≤ 0.01) the probability of smoking initiation with one puff, daily smoking initiation, and heavy smoking initiation among people younger than age 21 by 4, 4 and 2 percentage points, respectively; and among people age 21–30, the probability of smoking initiation with one puff (*p* ≤ 0.1), daily smoking initiation (*p* ≤ 0.01), and heavy smoking initiation (*p* ≤ 0.01) are reduced by 2–3 percentage points. The effects of SFA bars in laws with exemptions in reducing one-puff and daily initiation are larger among people younger than age 21 than people age 21 or older. This is not surprising since the age of smoking initiation in NLSY 97 peaks at age 18 and in the early study period when respondents were most susceptible to smoking initiation, most states only imposed SFA laws in bars with some exemptions. It was not until later years when more states increased the strengths of their laws.

Results in Table 3 regarding smoking relapse show that the 100% SFA law without exemption significantly reduce smoking relapse into daily smoking (*p* ≤ 0.05) and relapse into heavy smoking (*p* ≤ 0.01) by about 2 percentage points among people age 21 or older. In addition, SFA laws with some exemptions significantly reduce smoking relapse into non-daily smoking (*p* ≤ 0.1) by about 3 percentage points. The effects of laws appear no significant differences by the age 21 cutoff.

Table 3The effects of SFA in bars on smoking initiation_NLSY97, 1997–2009.

**Table:** 

	Initiation and Logistic Regressions			Relapse and Multinomial Regressions			
Variables	One-Puff	Daily	Heavy	Non-Daily	Daily	Light	Heavy
OR (95% CI). State and year fixed effects, SFA policy index are included as covariates							
Cigarette Tax	0.955	0.573 ^**^	1.523 ^*^	0.821	1.099	0.771^*^	1.245
	(0.649, 1.405)	(0.328, 1.000)	(0.931, 2.493)	(0.593, 1.137)	(0.642, 1.881)	(0.567, 1.049)	(0.606, 2.557)
SFA_bar_law	0.774 ^*^	0.320 ^***^	0.454 ^***^	0.795 *	1.099	0.923	0.988
	(0.596, 1.006)	(0.201, 0.510)	(0.248, 0.831)	(0.617, 1.024)	(0.798, 1.514)	(0.673, 1.266)	(0.620, 1.575)
SFA_bar_law × Age < 21	0.628 ^***^	0.699 ^**^	0.776	0.940	0.731	0.933	0.897
	(0.528, 0.748)	(0.525, 0.930)	(0.476, 1.264)	(0.801, 1.103)	(0.503, 1.064)	(0.786, 1.107)	(0.553, 1.455)
SFA_bar_ban	1.100	0.990	0.862	0.974	0.657 ^**^	1.017	0.533^***^
	(0.763, 1.584)	(0.655, 1.497)	(0.468, 1.587)	(0.831, 1.141)	(0.456, 0.947)	(0.812, 1.275)	(0.371, 0.767)
SFA_bar_ban × Age < 21	0.813	0.890	1.138	1.160	1.242	1.251^*^	0.680
	(0.448, 1.475)	(0.608, 1.302)	(0.562, 2.304)	(0.848, 1.587)	(0.706, 2.185)	(0.979, 1.599)	(0.135, 3.427)
N	38,988	48,271	50,366	27,001		27,927	

Note: Odds ratios are reported. 95% CI clustered at the state level are in parentheses. Other controls in Table 2 such as year and state fixed effects, gender, race, education, duration dependence, SFA laws in restaurants, PUP index, state spending on tobacco control programs and SFA index are not shown. ^*^ *p* ≤ 0.1, ^**^ *p* ≤ 0.05, ^***^ *p* ≤ 0.01.

**Table:** 

	Initiation and Logistic Regressions			Relapse and Multinomial Regressions			
Variables	One-Puff	Daily	Heavy	Non-daily	Daily	Light	Heavy
Marginal Effects (Standard errors). State and year fixed effects, SFA policy index are included as covariates							
SFA_bar_law × age < 21	−0.042 ^***^	−0.039 ^***^	−0.019 ^***^	−0.033 ^*^	−0.010	−0.019	−0.003
	(0.009)	(0.007)	(0.007)	(0.017)	(0.014)	(0.022)	(0.013)
SFA_bar_law × age ≥ 21	−0.015 ^*^	−0.030 ^***^	−0.015^***^	−0.029 ^*^	0.008	−0.011	0.0001
	(0.008)	(0.007)	(0.006)	(0.015)	(0.009)	(0.022)	(0.009)
SFA_bar_ban × age < 21	−0.007	−0.003	−0.0004	0.017	−0.013	0.040	−0.040
	(0.014)	(0.006)	(0.007)	(0.024)	(0.021)	(0.025)	(0.033)
SFA_bar_ban × age ≥ 21	0.006	−0.0003	−0.003	0.001	−0.024^**^	0.007	−0.024^***^
	(0.011)	(0.006)	(0.006)	(0.010)	(0.011)	(0.015)	(0.007)
N	38,988	48,271	50,366	27,001		27,927	

Note: Robust standard errors clustered at the state level are in parentheses. Other controls in Table 2 such as year and state fixed effects, gender, race, education, duration dependence, SFA laws in restaurants, PUP index, state spending on tobacco control programs and SFA index are not shown. ^*^ *p* ≤ 0.1, ^**^ *p* ≤ 0.05, ^***^ *p* ≤ 0.01.

**Table 4:** The effects of SFA in bars on smoking initiation_NLSY97, 1997–2009, sensitivity analysis.

Variables	Initiation and Logistic Regressions			Relapse and Multinomial Regressions			
	One-Puff	Daily	Heavy	Non-daily	Daily	Light	Heavy
OR (95% CI). State fixed effects, year trend, and SFA policy index are included as covariates							
Cigarette Tax	0.875	0.558 ^**^	1.246	0.771 ^*^	1.112	0.728 ^**^	1.261
	(0.540, 1.419)	(0.335, 0.931)	(0.634, 2.449)	(0.592, 1.004)	(0.659, 1.877)	(0.558, 0.949)	(0.633, 2.510)
SFA_bar_law	0.722 ^**^	0.315 ^***^	0.406 ^***^	0.804 ^*^	1.097	0.922	0.983
	(0.540, 0.965)	(0.202, 0.490)	(0.216, 0.762)	(0.622, 1.039)	(0.779, 1.544)	(0.681, 1.248)	(0.616, 1.568)
SFA_bar_law × Age < 21	0.643 ^***^	0.688 ^**^	0.764	0.929	0.701 ^*^	0.923	0.886
	(0.542, 0.762)	(0.514, 0.920)	(0.415, 1.409)	(0.808, 1.069)	(0.468, 1.050)	(0.787, 1.083)	(0.547, 1.434)
SFA_bar_ban	1.061	0.961	0.788	0.973	0.647 ^**^	1.014	0.526 ^***^
	(0.687, 1.638)	(0.603, 1.531)	(0.399, 1.556)	(0.832, 1.137)	(0.442, 0.945)	(0.808, 1.273)	(0.369, 0.751)
SFA_bar_ban × Age < 21	0.979	1.080	1.540	1.179	1.222	1.314 ^**^	0.673
	(0.524, 1.831)	(0.742, 1.573)	(0.746, 3.178)	(0.891, 1.562)	(0.686, 2.177)	(1.060, 1.630)	(0.139, 3.261)
OR (95% CI). State fixed effects, year trend, and individual SFA policy variables are included as covariates							
Cigarette Tax	0.879	0.576 ^**^	1.133	0.806	1.087	0.775 ^*^	1.213
	(0.533, 1.449)	(0.341, 0.974)	(0.554, 2.319)	(0.602, 1.078)	(0.621, 1.903)	(0.563, 1.013)	(0.578, 2.546)
SFA_bar_law	0.706	0.230 ^***^	0.184 ^***^	0.666 ^***^	1.026	0.803	0.805
	(0.461, 1.081)	(0.087, 0.610)	(0.102, 0.333)	(0.507, 0.874)	(0.744, 1.416)	(0.501, 1.287)	(0.540, 1.201)
SFA_bar_law × Age < 21	0.643 ^***^	0.681 ^**^	0.772	0.927	0.700 ^*^	0.926	0.876
	(0.537, 0.770)	(0.502, 0.923)	(0.414, 1.441)	(0.814, 1.057)	(0.470, 1.043)	(0.796, 1.077)	(0.540, 1.423)
SFA_bar_ban	1.149	1.221	0.824	0.968	0.682 ^*^	0.957	0.621 ^*^
	(0.766, 1.724)	(0.686, 2.173)	(0.425, 1.599)	(0.762, 1.230)	(0.449, 1.037)	(0.731, 1.254)	(0.366, 1.053)
SFA_bar_ban × Age < 21	0.875	1.002	1.321	1.205	1.362	1.412 ^***^	0.711
	(0.449, 1.703)	(0.693, 1.449)	(0.686, 2.544)	(0.947, 1.534)	(0.747, 2.486)	(1.174, 1.698)	(0.153, 3.294)
N	38,988	48,271	50,366	27,001		27,927	

Note: Odds ratios are reported. 95% CI clustered at the state level are in parentheses. Other controls in Table 2 such as gender, race, education, duration dependence, SFA laws in restaurants, PUP index, state spending on tobacco control programs and SFA index are not shown. ^*^ *p* ≤ 0.1, ^**^ *p* ≤ 0.05, ^***^ *p* ≤ 0.01.

Sensitivity analyses by using a linear year trend instead of year fixed effects and by using a linear year trend and individual SFA policy scores instead of the average SFA policy index are shown in Table 4. The estimates are very close to those results in Table 3. In addition, although not shown in the paper, we also conducted all regressions by restricting samples to respondents aged 21 or older and by restricting samples to respondents with non-missing covariates. The results are consistent. Table 4 also suggests that, consistent with previous studies [8,9,10,11], higher cigarette taxes significantly reduce daily smoking initiation and relapsing to nondaily or light smoking.

## 4. Conclusions

This study examines the impacts of SFA laws in bars on smoking initiation/relapse using NLSY97 data and provides important evidence on the effectiveness of SFA laws in deterring smoking initiation and progression. We find that SFA laws in bars with some exemptions significantly reduce (*p* ≤ 0.01) the probability of smoking initiation with one puff, daily smoking initiation, and heavy smoking initiation by 2–4 percentage points and the effects in reducing one-puff and daily initiation are larger among people younger than age 21 than people age 21 or older. This law also significantly reduces smoking relapse into non-daily smoking. In addition, the 100% SFA law without exemptions significantly reduce smoking relapse into daily smoking (*p* ≤ 0.05) and relapse into heavy smoking (*p* ≤ 0.01) by about 2 percentage points among people age 21 or older. The interaction terms of laws and the age indicator suggest that, while the reduction in initiation due to SFA laws in bars is larger for people age 20 or younger than for people over 20, the reduction in relapse in these two age groups does not differ. Consistent with the literature, we also find that higher cigarette taxes significantly reduce (*p* ≤ 0.05) daily smoking initiation and relapse into nondaily (*p* ≤ 0.1) or light smoking (*p* ≤ 0.05).

This study is subject to several limitations: first, this study does not control for local SFA laws, which may be stronger than state laws; second, smokers who had already initiated daily or heavy smoking but did not smoke daily or heavily in the baseline year 1997 cannot be identified; and third, the results using non-missing longitudinal samples over time cannot be generalized to the population level. Nevertheless, this study utilizes longitudinal data that spans more than 10 years and provides important information to policy makers about the effectiveness of SFA laws in bars in deterring smoking initiation and escalation into daily/heavy smoking. This evidence, along with the existing evidence that SFA laws help reduce secondhand smoke exposure and drinking, shows that the positive public health impact of SFA laws may be very significant. Future research should consider including respondents’ drinking status and model the simultaneous decisions of smoking and binge drinking in response to policies aimed at inhibiting these two risk behaviors.
